# Description of the first fungal dye-decolorizing peroxidase oxidizing manganese(II)

**DOI:** 10.1007/s00253-015-6665-3

**Published:** 2015-05-13

**Authors:** Elena Fernández-Fueyo, Dolores Linde, David Almendral, María F. López-Lucendo, Francisco J. Ruiz-Dueñas, Angel T. Martínez

**Affiliations:** Centro de Investigaciones Biológicas, CSIC, Ramiro de Maeztu 9, E-28040 Madrid, Spain

**Keywords:** *Pleurotus ostreatus*, Dye-decolorizing peroxidases, Genome mining, Lignocellulose secretome, Manganese(II) oxidation, Evolutionary history

## Abstract

Two phylogenetically divergent genes of the new family of dye-decolorizing peroxidases (DyPs) were found during comparison of the four DyP genes identified in the *Pleurotus ostreatus* genome with over 200 DyP genes from other basidiomycete genomes. The heterologously expressed enzymes (*Pleos*-DyP1 and *Pleos*-DyP4, following the genome nomenclature) efficiently oxidize anthraquinoid dyes (such as Reactive Blue 19), which are characteristic DyP substrates, as well as low redox-potential dyes (such as 2,2-azinobis-(3-ethylbenzothiazoline-6-sulfonic acid)) and substituted phenols. However, only *Pleos*-DyP4 oxidizes the high redox-potential dye Reactive Black 5, at the same time that it displays high thermal and pH stability. Unexpectedly, both enzymes also oxidize Mn^2+^ to Mn^3+^, albeit with very different catalytic efficiencies. *Pleos*-DyP4 presents a Mn^2+^ turnover (56 s^−1^) nearly in the same order of the two other Mn^2+^-oxidizing peroxidase families identified in the *P. ostreatus* genome: manganese peroxidases (100 s^−1^ average turnover) and versatile peroxidases (145 s^−1^ average turnover), whose genes were also heterologously expressed. Oxidation of Mn^2+^ has been reported for an *Amycolatopsis* DyP (24 s^−1^) and claimed for other bacterial DyPs, albeit with lower activities, but this is the first time that Mn^2+^ oxidation is reported for a fungal DyP. Interestingly, *Pleos*-DyP4 (together with ligninolytic peroxidases) is detected in the secretome of *P. ostreatus* grown on different lignocellulosic substrates. It is suggested that generation of Mn^3+^ oxidizers plays a role in the *P. ostreatus* white-rot lifestyle since three different families of Mn^2+^-oxidizing peroxidase genes are present in its genome being expressed during lignocellulose degradation.

**Electronic supplementary material:**

The online version of this article (doi:10.1007/s00253-015-6665-3) contains supplementary material, which is available to authorized users.

## Introduction

Peroxidases (EC1.11.1) represent a large group of oxidoreductases that use hydrogen peroxide as the final electron acceptor. Heme peroxidases were classified by Welinder (1992) into two superfamilies, animal and nonanimal peroxidases, the second one being divided into three classes according to their origin. Class I peroxidases come from the prokaryotic lineage, and classes II and III are secretory peroxidases from fungi and plants, respectively. The discovery of new peroxidase types in the last few years resulted in two novel (super)families of secreted fungal peroxidases: hemethiolate peroxidases (HTP), in which chloroperoxidases (EC 1.11.1.10) and unspecific peroxygenases (EC 1.11.2.1) are included, and dye-decolorizing peroxidases (DyPs; EC 1.11.1.19) (Hofrichter et al. 2010; Ruiz-Dueñas and Martínez 2010; Sugano et al. 2007; Sugano 2009).

DyPs constitute a group of enzymes phylogenetically unrelated to the catalase-peroxidase superfamily, where the classical nonanimal peroxidases—including lignin peroxidase (LiP; 1.11.1.14), manganese peroxidase (MnP; EC 1.11.1.13), and versatile peroxidase (VP; EC 1.11.1.16)—are currently classified (Zámocký et al. 2015). From a structural point of view, DyPs are members of the functionally diverse CDE superfamily (including chlorite dismutases, DyP-type enzymes, and proteins related to *Escherichia coli* EfeB) characterized by the presence of a ferredoxin-like core of β-sheets (Goblirsch et al. 2011). The first DyP was discovered in a culture of the fungus *Bjerkandera adusta* (initially described as *Geotrichum candidum*) (Kim and Shoda 1999; Sugano et al. 1999). Despite only seven fungal and eleven bacterial DyPs have been purified and characterized to date (Linde et al. 2015b), 233 DyP sequences from fungal, bacterial and archaeal genomes, and other sources are deposited in PeroxiBase (http://peroxibase.toulouse.inra.fr; March 2015 data), confirming their ubiquitous distribution (Colpa et al. 2014; Sugano 2009).

The range of biological processes in which peroxidases are involved includes defense, immune response, pathogenicity, detoxification, and biomass degradation (Torres and Ayala 2010). The natural ecophysiological role of DyPs still remains unclear, but these enzymes offer attractive properties for biotechnological purposes, being able to oxidize anthraquinoid and other dye types (Kim and Shoda 1999; Liers et al. 2010), carotenoids (Scheibner et al. 2008; Zelena et al. 2009a), and phenolic compounds (Liers et al. 2014; Linde et al. 2014; Sugano et al. 2000), among others. Moreover, the ability of DyPs to oxidize lignin-related compounds has been reported (Ahmad et al. 2011; Brown et al. 2012; Roberts et al. 2011; Salvachúa et al. 2013), although their catalytic efficiency on veratryl alcohol (Liers et al. 2013; Salvachúa et al. 2013) and nonphenolic lignin model dimers (Liers et al. 2010) is very low compared with that of basidiomycete LiPs (Linde et al. 2015b).

According to genomic data, LiPs, MnPs, and VPs are exclusive of typical white-rot (ligninolytic) basidiomycetes (most of them in the order *Polyporales*) (Floudas et al. 2012, 2015; Ruiz-Dueñas et al. 2013). Even though DyP genes do not show a so clear distribution, they are significantly more abundant in the genomes of lignin-degrading basidiomycetes (white-rot fungi) than in those of cellulose-degrading basidiomycetes (brown-rot fungi) according to the above genomic studies. This fact, together with their ability to oxidize aromatic compounds, suggests that DyPs could be involved in degradation of lignin compounds and derived soil organic matter. Moreover, the ability to oxidize Mn^2+^, a typical substrate of MnPs and VPs (Ruiz-Dueñas et al. 2009), has been reported for bacterial DyPs from *Rhodococcus josti*, *Amycolatopsis* sp., and *Pseudomonas fluorescens* (Brown et al. 2012; Rahmanpour and Bugg 2015; Roberts et al. 2011). This pointed to possible involvement in lignin degradation via Mn^3+^-initiated lipid peroxidation reactions, as proposed for MnP (Bao et al. 1994).

*Pleurotus ostreatus*, a white-rot fungus in the order *Agaricales*, is the second most consumed edible mushroom worldwide (Sánchez 2010). Its genome includes a total of 17 peroxidase genes: nine class II peroxidase genes (of the MnP and VP families) and one class I peroxidase gene, as well as four and three genes from the DyP and HTP (super)families, respectively (Ruiz-Dueñas et al. 2011). The catalytic properties of *P. ostreatus* VPs and MnPs have been thoroughly investigated after heterologous expression, and their secretion in lignocellulose media has been confirmed (Fernández-Fueyo et al. 2014b, d). However, a single report on *P. ostreatus* DyPs has been published to date, describing the *Pleos*-DyP1-encoding gene (Faraco et al. 2007).

In the present study, we identified the DyPs and other peroxidases secreted by *P. ostreatus* when grown on lignocellulosic substrates by using nanoflow liquid chromatography coupled to tandem mass spectrometry (nLC-MS/MS). Simultaneously, four DyP genes were identified in the *P. ostreatus* genome and two of them (presenting different evolutionary histories) were heterologously expressed in *E. coli* as active soluble enzymes, and their catalytic properties compared with two VPs and two MnPs from the same fungus. Unexpectedly, these *P. ostreatus* DyPs were able to oxidize Mn^2+^, a characteristic that had not been reported before for any other fungal DyPs.

## Material and methods

### *P. ostreatus* strains and genome

Dikaryotic *P. ostreatus* N001 (CECT20600), maintained in 2 % malt extract agar (MEA), was used in this study. The genomic sequences of monokaryons PC9 (CECT20311) and PC15 (CECT20312), isolated from the above dikaryon (Larraya et al. 1999), are available (http://genome.jgi-psf.org/PleosPC9_1 and http://genome.jgi-psf.org/PleosPC15_2). They were sequenced in a JGI project coordinated by A.G. Pisabarro (Public University of Navarre, Spain).

### DyP screening in the *P. ostreatus* genome

The inventory of DyP coding genes in the *P. ostreatus* genome was obtained by the following: (i) screening the automatically annotated genomes; (ii) revising and manually curating the positions of introns and the C- and N-termini, the latter using SignalP 4.1 for predicting signal peptides; (iii) comparing the predicted amino acid sequences with related peroxidases, after multiple alignment with MEGA5 (Tamura et al. 2011); and (iv) confirming the presence of characteristic residues at the heme pocket and substrate oxidation sites. These residues were identified after homology modeling at the SWISS-MODEL server using the crystal structures of *Auricularia auricula*-*judae* (PDB 4W7J) and *B. adusta* (PDB 2D3Q) DyPs as templates. Finally, the revised sequences were compared with all the DyP sequences (a total of 218) from the 114 basidiomycete (*Agaricomycotina*) genomes currently (7 December 2014) sequenced and annotated at JGI, which are available at the MycoCosm portal (http://genome.jgi-psf.org/programs/fungi). A phylogram—also including available GenBank sequences from DyPs of *A. auricula*-*judae* (JQ650250), *Mycetinis scorodonius* (CS490662 and CS490657), *Ganoderma lucidum* (ADN05763), *Termitomyces albuminosus* (AAM21606), and an unidentified *Polyporaceae* species (AAB58908)—was constructed with MEGA5 using MUSCLE  alignment and maximal likelihood clustering.

### Gene synthesis

The mature protein coding sequences of two DyP (JGI protein ID 62271 and 1069077 from PC15, corresponding to *Pleos*-DyP1 and *Pleos*-DyP4, respectively), two MnP (1089546 and 1041740 from PC15, corresponding to *Pleos*-MnP3 and *Pleos*-MnP6, respectively), and two VP genes (137757 from PC9, corresponding to *Pleos*-VP1 and 1113241 from PC15, corresponding to *Pleos*-VP2) from the *P. ostreatus* genomes (http://genome.jgi-psf.org/PleosPC9_1 and http://genome.jgi-psf.org/PleosPC15_2) were synthesized by ATG:biosynthetics (Merzhausen, Germany), after verifying that all the codons had previously been used for expressing other genes in the *E. coli* strains mentioned below, and substituting them when required. The optimized cDNA sequences of *Pleos*-DyP1 (KP973935), *Pleos*-DyP4 (KP973936), *Pleos*-MnP3 (KP973939), *Pleos*-MnP6 (KP973940), *Pleos*-VP1 (KP973937), and *Pleos*-VP2 (KP973938) were deposited at GenBank.

### DyP production in *E. coli*

The above two DyP coding sequences were cloned in the pET23a vector (Novagen, Darmstadt, Germany), and the resulting plasmids (pET23a-62271 and pET23a-1069077) were used for expression in *E. coli* BL21(DE3)pLysS (Novagen, Darmstadt, Germany). Cells were grown for 3 h at 37 °C in Terrific Broth, induced with 1 mM isopropyl-β-d-thiogalactopyranoside (IPTG) and grown further for 48 h at 16 °C in the presence of 20 μM of hemin. Cells were harvested by centrifugation at 8000 rpm for 10 min at 4 °C. Bacteria were resuspended in 300 mL lysis buffer (20 mM Tris-HCl pH 8.0 containing 1 mM EDTA, and 5 mM DTT), supplemented with lysozyme (Sigma-Aldrich, Steinheim, Germany) (2 mg.mL^−1^) and DNase I (Roche Diagnostics, Mannheim, Germany). After 1 h of incubation, cells were sonicated, and debris was removed by centrifugation at 20,000 rpm for 4 h. The supernatants were concentrated with Amicon (Millipore, Darmstadt, Germany) of 10-kDa cutoff, and dialyzed against 20 mM acetate, pH 4.3. Insoluble material was eliminated (15,000 rpm for 30 min) and the solution further dialyzed (10 mM Tris, pH 7.5).

DyPs were purified using an ÄKTA high-performance liquid chromatography (HPLC) system (GE Healthcare Bio-Sciences AB, Uppsala, Sweden), in three consecutive steps. The first separation was performed on a 6-mL Resource™ Q cartridge (GE Healthcare Bio-Sciences AB, Uppsala, Sweden) using 10 mM Tris (pH 7.5) at a flow rate of 2 mL.min^−1^. After 15 mL, the retained proteins were eluted with a 0–0.35 M NaCl gradient in 50 mL, followed by 0.5–1 M NaCl gradient in 10 mL, and 1 M NaCl in 15 mL. Peroxidase activity was followed by 2,2-azinobis-(3-ethylbenzothiazoline-6-sulfonic acid) (ABTS) oxidation in the presence of H_2_O_2_, as described below. The appropriate fractions were pooled, concentrated, and dialyzed against 10 mM Tris (pH 7.5), and loaded into a Mono Q high-resolution 5/5 column (GE Healthcare Bio-Sciences AB, Uppsala, Sweden) at a flow rate of 1 mL.min^−1^ (same buffer) using a 0–0.3 M NaCl gradient in 40 mL, followed by 1 M NaCl in 10 mL. Finally, size-exclusion chromatography in a Superdex-75 HR 10/30 column (GE Healthcare Bio-Sciences AB, Uppsala, Sweden) with 10 mM tartrate (pH 5), containing 100 mM NaCl, at 0.2 mL.min^−1^, was performed. *Pleos*-DyP1 and *Pleos*-DyP4 purification was confirmed by sodium dodecyl sulfate-polyacrylamide gel electrophoresis (SDS-PAGE) in 12 % gels stained with Coomassie Brilliant Blue R-250 (Sigma). Electronic absorption spectra of the purified enzymes were recorded with an Agilent 8453 (Agilent Technologies, Santa Clara, USA) diode array UV-visible spectrophotometer.

### MnP and VP production in *E. coli*

The *Pleos*-MnP3 (PC15 1089546), *Pleos*-MnP6 (PC15 1041740), *Pleos*-VP1 (PC9 137757), and *Pleos*-VP2 (PC15 1113241) coding sequences were cloned in the pFLAG1 (International Biotechnologies Inc., Kodak, CT, USA) or pET23a (+) (Novagen, Darmstadt, Germany) vectors, and the resulting plasmids (pFLAG1-1089546, pET23a-1041740, pFLAG1-137757, and pFLAG1-1113241) were used for expression in *E. coli* K-12 W3110 (pFLAG1 plasmids) and BL21(DE3)pLysS (pET23a plasmids). Cells were grown for 3 h in Terrific Broth, induced with 1 mM IPTG, and grown further for 4 h. The apoenzyme accumulated in inclusion bodies, as observed by SDS-PAGE, and was solubilized with 8 M urea.

In vitro refolding was performed using 0.16 M urea, 5 mM Ca^2+^, 20 μM hemin, 0.5 mM oxidized glutathione, 0.1 mM dithiothreitol, and 0.1 mg.mL^−1^ protein, at pH 9.5 (Pérez-Boada et al. 2002). For *Pleos*-MnP6 optimized refolding was obtained using 0.1 M urea, 5 mM Ca^2+^, 20 μM hemin, 1.5 mM oxidized glutathione, 0.1 mM dithiothreitol, and 0.1 mg.mL^−1^ protein, at pH 8. Enzymes were purified by Resource-Q chromatography using a 0–0.3 M NaCl gradient (2 mL.min^−1^, 20 min) in 10 mM tartrate (pH 5.5) containing 1 mM CaCl_2_.

### Peroxidase kinetic constants and optimal pH

The optimal pH for substrate oxidation by the two *P. ostreatus* DyPs was determined using saturating concentrations of (i) Reactive Black 5 (RB5; 15 μM), Reactive Blue 19 (RB19; 200 μM), ABTS (5 mM), and 2,6-dimethoxyphenol (DMP; 60 mM) in 0.1 M Britton-Robinson (B&R) buffer (pH 2–5.5); and (ii) Mn^2+^ (6 mM) in 0.1 mM tartrate buffer (pH 2–6), and the assays described below.

The kinetic constants of the *P. ostreatus* DyPs, MnPs, and VPs were estimated from the absorbance changes observed during substrate oxidation in 0.1 M tartrate (at optimal pH values) at 25 °C in a Thermo BioMate 5 spectrophotometer (Thermo Fisher Scientific, Waltham, USA). The reactions were initiated by addition of H_2_O_2_ to a final concentration of 0.25 mM. Oxidation of Mn^2+^ was followed at pH 4.5 by monitoring formation of Mn^3+^-tartrate complex (*ε*_238_ 6.5 mM^−1^.cm^−1^). RB19 and ABTS oxidation were assayed at pH 3.5 and monitored by RB19 disappearance (*ε*_595_ 10 mM^−1^.cm^−1^) and formation of ABTS cation radical (*ε*_436_ 29.3 mM^−1^.cm^−1^). RB5 and DMP oxidation were assayed at pH 3 and monitored by RB5 disappearance (*ε*_598_ 30 mM^−1^.cm^−1^) and dimeric coerulignone (*ε*_469_ 55 mM^−1^.cm^−1^) formation, respectively. Means and standard errors for Michaelis constant (*K*_m_) and enzyme turnover (*k*_cat_) values were obtained by nonlinear least-squares fitting to the Michaelis-Menten model. Fitting of these constants to the normalized equation *v* = (*k*_cat_/*K*_m_)[*S*]/(1 + [*S*]/*K*_m_) yielded the catalytic efficiency values (*k*_cat_/*K*_m_) with their corresponding standard errors.

### Studies on DyP pH and thermal inactivation

To study the effect of incubation at different pH values on the activity of the two *P. ostreatus* DyPs (0.01 μM), the enzymes were dissolved in 0.1 M B&R buffer in the range of pH 2–9, and kept at 4 °C for different time periods. Activity was determined by oxidation of ABTS (5 mM) under the conditions described above. Residual activities were measured after 1 min (to evaluate the initial survival of the enzyme at each pH value) and 1, 4, 24 and 120 h incubation. The highest activity after 1 min (at any pH) was taken as 100 % activity, and the percentages of residual activity at the different times and pH conditions were calculated according to this maximal value.

In a similar way, to study the effect of incubation at different temperatures, the enzymes in 10 mM tartrate (pH 5) were kept for 10 min in the temperature range of 25–85 °C. Residual activity was determined at 25 °C, as described above, and that obtained after 25 °C incubation was taken as 100 %. Temperature stability was finally presented as 10-min *T*_50_ value, i.e., that temperature at which 50 % of the activity was lost after incubation for the above time period.

### Secretomic analyses of *P. ostreatus* peroxidases

Secretomic studies were performed with dikaryotic *P. ostreatus* N001 in glucose and (two different) lignocellulose culture media. Glucose cultures were grown in 1 L shaken (200 rpm) flasks with 200 mL of HAT medium (Spinnler et al. 1994) containing 10 g glucose, 0.2 g KH_2_PO_4_, 0.5 g MgSO_4_·7H_2_O, 1 g casamino acids, 1 g yeast extract, 0.368 g ammonium tartrate, and 1 L of distilled water. Inocula consisted of 15 mL of homogenized actively growing mycelium from M7GY (Castanera et al. 2012) liquid cultures incubated at 200 rpm. Lignocellulose cultures were grown on 10 g of milled wheat straw or small poplar chips (particle size < 4 mm) soaked with 70 mL of distilled water in 1 L flasks (stationary cultures). Inocula consisted of 15 mL of homogenized mycelium from M7GY cultures. All the above cultures were maintained at 25 °C.

Total extracellular proteins in the filtrates of cultures grown for 21 days in the above media were freeze-dried, resuspended in 10 mM tartrate (pH 5), impurities removed by a short PAGE run, and stained by Colloidal Blue Kit (Invitrogen, Thermo Fisher Scientific, Waltham, USA). The protein band was cut and destained using 50 mM ammonium bicarbonate in 50 % acetonitrile (ACN), reduced with 10 mM dithiothreitol for 30 min at 56 °C, alkylated with 55 mM iodoacetamide in the dark for 30 min at 24 °C, and digested with 12.5 ng.μL^−1^ trypsin in 50 mm ammonium bicarbonate, overnight at 30 °C. Peptides were extracted at 37 °C using 100 % ACN and then 0.5 % trifluoroacetic acid, dried, cleaned using ZipTip with 0.6 μL C18 resin (Millipore), and reconstituted in 5 μL of 0.1 % formic acid in 2 % ACN.

Tryptic peptides were analyzed in an LTQ-Orbitrap Velos mass spectrometer (Thermo Fisher Scientific, Waltham, USA) coupled to a nanoEasy HPLC equipment (Proxeon). Peptides were first trapped onto a C18-A1 ASY-Column 2 cm precolumn (Thermo Fisher Scientific, Waltham, USA) and then eluted onto a Biosphere C18 column (75 μm inner diameter, 15 cm long, and 3 μm particle size) (NanoSeparations) using a 130-min gradient from 0–45 % buffer-B (buffer-A: 0.1 % formic acid in 2 % ACN; buffer B: 0.1 % formic acid in pure ACN) at a flow rate of 250 nL.min^−1^. Mass spectra were acquired in the positive ion mode and data-dependent manner selecting the 20 most intense ions for fragmentation using collision-induced dissociation (CID). MS spectra (*m*/*z* 300–1600) were acquired in the Orbitrap with a target value of 1,000,000 at a resolution of 30,000 (at *m*/*z* 400), and MS2 spectra were acquired in the linear ion trap with a target value of 10,000 and normalized collision energy of 35 %. Precursor ion charge state screening and monoisotopic precursor selection were enabled. Singly charged ions and unassigned charge states were rejected. Dynamic exclusion was enabled with a repeat count of 1 and exclusion duration of 30 s.

Acquired spectra were searched against the *P. ostreatus* PC9 and PC15 genomic databases, downloaded from JGI (http://genome.jgi-psf.org/programs/fungi), using Sequest search engine through Proteome Discoverer (version 1.4). As for the search parameters, precursor and fragment mass tolerance were set to 10 ppm and 0.8 Da, respectively. Carbamidomethylation of cysteines was set as a fixed modification, and oxidation of methionines was set as a dynamic modification. Two missed cleavages were allowed. Identified peptides were validated using Percolator algorithm with a *q* value threshold of 0.01.

## Results

### DyP gene analysis in the *P. ostreatus* genome

**Fig. 1:**
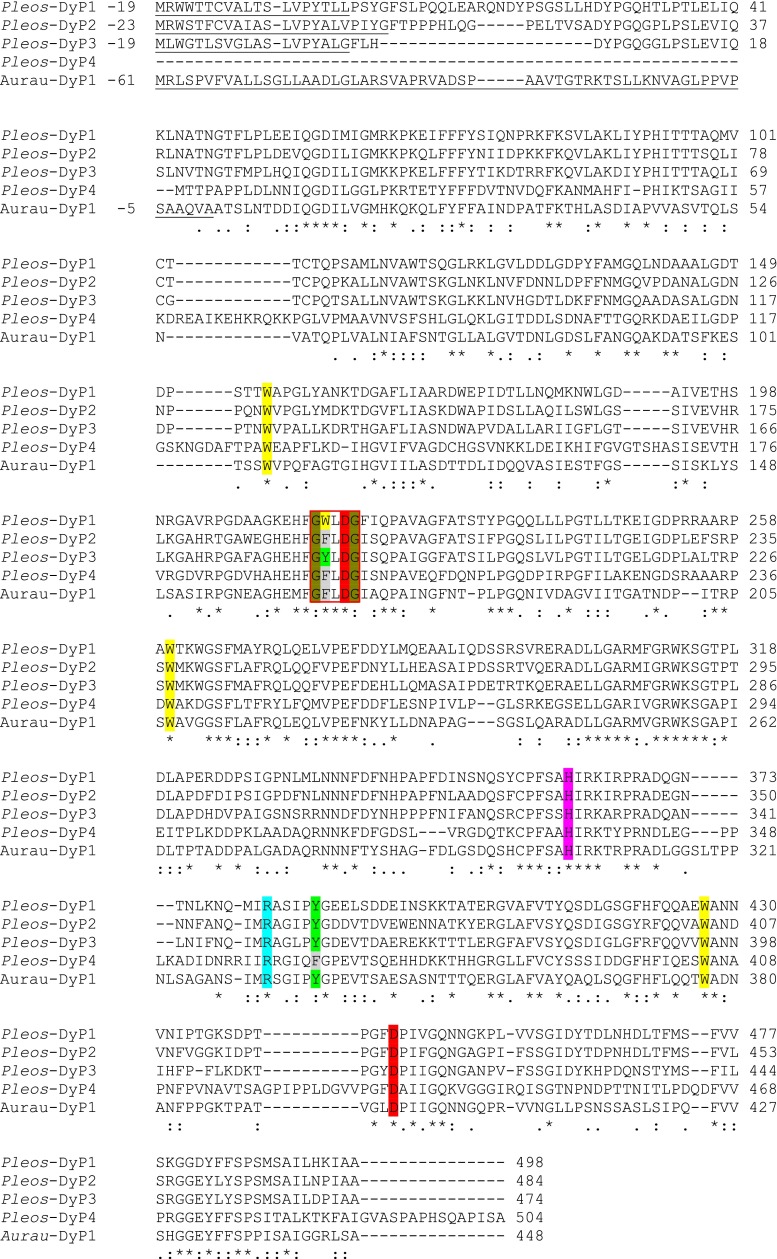
Alignment of the four DyPs from the *P. ostreatus* genome (*Pleos*-DyP1 to *Pleos*-DyP4; JGI protein ID 62271, 1092668, 52170, and 1069077, respectively) and the best known *A. auricula*-*judae* DyP (*Aurau*-DyP1, GenBank JQ650250). Numbering (*right*) corresponds to the predicted mature proteins (numbering of underlined signal peptide, when present, is indicated on the left). Highlighted residues include the following: (i) proximal histidine (*magenta*) and aspartic acid (*red*); (ii) distal-side arginine (*cyan*) and aspartic acid (*red*), the latter forming part of the GXXDG motif (*red box*) together with two conserved glycines (*olive*), a leucine, and an aromatic residue (*two positions before*) corresponding to a phenylalanine (*gray*) that is substituted by tryptophan (*yellow*) or tyrosine (*green*) in two of the sequences; and (iii) four aromatic residues exposed to the solvent, corresponding to three conserved tryptophans (*yellow*), and one tyrosine (*green*) that is substituted by phenylalanine (*gray*) in one of the sequences. Alignment was produced with ClustalW2 (European Bioinformatics Institute, Hinxton, UK), and *symbols below the sequences* indicate full conservation of the same (*asterisk*) or equivalent residues (*colon*) and partial residue conservation (*dot*)

Four DyP coding genes were identified and manually annotated in the genomes of two *P. ostreatus* monokaryons (PC9 and PC15). The alleles from these two monokaryons are 99.0 % (*Pleos*-DyP1), 99.8 % (*Pleos*-DyP2), 98.7 % (*Pleos*-DyP3), and 98.2 % (*Pleos*-DyP4) identical at the protein level, and the gene models from the genome of *P. ostreatus* PC15 v2.0 (http://genome.jgi-psf.org/PleosPC15_2) are analyzed here. *Pleos*-DyP1 (protein ID 62271, scaffold 09:213879-216064), *Pleos*-DyP2 (protein ID 1092668, scaffold 04:657069-659105), and *Pleos*-DyP3 (protein ID 52170, scaffold 07:3196067-3198039) are constituted by ten translated exons giving rise to proteins of 516, 511, and 493 total amino acids, respectively, while *Pleos*-DyP4 (protein ID 1069077, scaffold 11:2524348-2526415) is formed by only eight exons that codify a 504 amino acid protein (small introns of 50–89 nucleotides separate the above exons in the four genes). Signal peptides of 19 amino acids in *Pleos*-DyP1 and *Pleos*-DyP3 (we manually curated the latter gene at the JGI portal to include this signal peptide), and 23 amino acids in *Pleos*-DyP2 were identified (no signal peptide was predicted for *Pleos*-DyP4) resulting in mature sequences of less than 500 amino acids for *Pleos*-DyP1 (498), *Pleos*-DyP2 (488), and *Pleos*-DyP3 (474) while mature *Pleos*-DyP4 would be formed by 504 amino acids (Fig. 1). The first three enzymes showed 63–74 % sequence identity among them (from 280–352 aligned residues) (Table S1 in the Supplementary Material). However, their individual sequence identities with *Pleos*-DyP4 were always below 40 % (and only 176–191 residues could be aligned) suggesting a divergent evolutionary origin of the latter protein.

### DyP molecular models

For the structural-functional classification of the *P. ostreatus* peroxidases, molecular models of the deduced mature proteins were prepared using related crystal structures as templates. These models confirmed the presence of important residues for DyP catalysis (Linde et al. 2015b) including as follows: (i) proximal histidine (His-361 and His-334 in *Pleos*-DyP1 and *Pleos*-DyP4, respectively) acting as the fifth ligand of the heme iron and H-bonded aspartic acid (Asp-445 and Asp-433 in *Pleos*-DyP1 and *Pleos*-DyP4, respectively); and (ii) second aspartic acid (Asp-218 and Asp-196 in *Pleos*-DyP1 and *Pleos*-DyP4, respectively), forming a part of a conserved GXXDG motif (Colpa et al. 2014), and neighbor arginine (Arg-382 and Arg-360 in *Pleos*-DyP1 and *Pleos*-DyP4, respectively) at the opposite side of the heme, helping the heterolytic cleavage of H_2_O_2_ to form compound I (the above residues are marked on the Fig. 1 alignment, together with other relevant residues mentioned below).

**Fig. 2:**
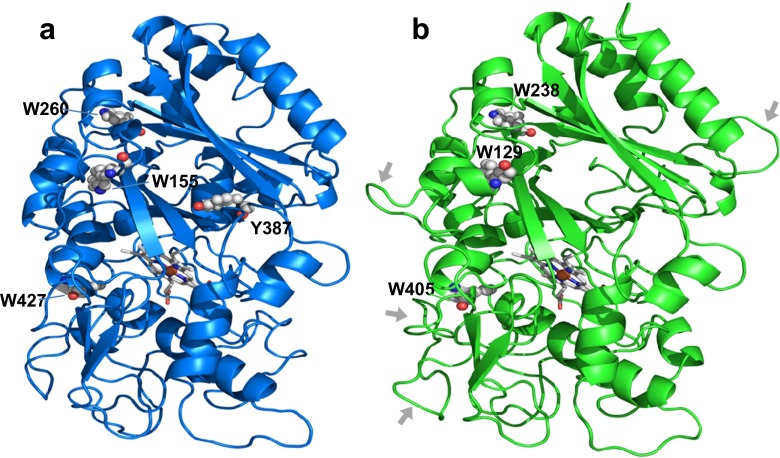
Molecular models of *Pleos*-DyP1 (**a**) and *Pleos*-DyP4 (**b**). The ribbon representations include 3–4 exposed aromatic residues (as *CPK*-*colored spheres*), the heme cofactor (as *CPK*-*colored sticks*), and some extra loops in *Pleos*-DyP4 (*arrows* in **b**). The two enzymes were modeled using related crystal structures as template (some N- and C-terminal residues are not included in the models)

The *Pleos*-DyP4 molecular model showed several significant differences with respect to the other three DyP models. They are illustrated in Fig. 2 showing the whole molecular models for *Pleos*-DyP1 and *Pleos*-DyP4. The *Pleos*-DyP4 molecular model (Fig. 2a) shows four extra loops (arrows) that, together with the 15-residue longer C-terminal tail (Fig. 2b), result in a longer mature protein, despite its 42-residue shorter N-terminal tail. Four aromatic residues are exposed to the solvent in *Pleos*-DyP1 (Fig. 2a) including Trp-427, homologous to *A. auricula*-*judae* catalytic Trp-377 (Linde et al. 2015a), together with two other tryptophans and one surface tyrosine. The latter residue is substituted by a phenylalanine in *Pleos*-DyP4 that, however, conserves the putative catalytic Trp-405 and the two other tryptophan residues (Fig. 2b). The same surface aromatic residues found in *Pleos*-DyP1 are present in *Pleos*-DyP2 and *Pleos*-DyP3 (Fig. 1).

After annotating the DyP-encoding genes in the *P. ostreatus* genome, and analyzing the predicted sequences and molecular structures of the corresponding enzymes, two of them (*Pleos*-DyP1 and *Pleos*-DyP4) were selected for heterologous expression and biochemical characterization, due to their divergent evolutionary origin.

### DyP production, purification, and electronic absorption spectra

**Fig. 3:**
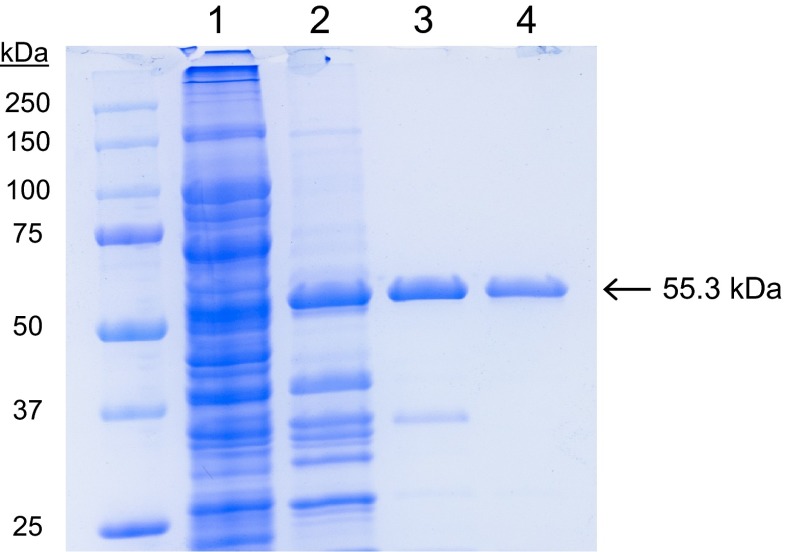
SDS-PAGE during purification of *Pleos*-DyP1 (55.3 kDa) to attain electrophoretic homogeneity. *Lanes 1* to *4* correspond to the whole soluble fraction from *E. coli* culture and protein samples after Resource Q, Mono Q, and Sephadex 75 purification steps (molecular mass standards are included)

For heterologous expression and purification, large-scale (9 L) cultures of *E. coli* transformants were grown under conditions resulting in *Pleos*-DyP1 and *Pleos*-DyP4 production as active proteins. For enzyme recovery, cells were lysed, centrifuged, and the supernatants concentrated and dialyzed. The purification process required three different chromatographic steps. As illustrated for *Pleos*-DyP4 (Fig. 3), SDS-PAGE monitoring of the purification process showed that the whole soluble fraction from the *E. coli* cultures (*lane 1*) was considerably enriched in the DyP (55.3 kDa) band by Resource Q chromatography (*lane 2*), but a number of other proteins remained. Most of these contaminating proteins were removed by Mono Q (*lane 3*), and, using a final Sephadex 75 step, electrophoretically homogeneous DyP was obtained (*lane 4*) with purification yields of 6.3 mg *Pleos*-DyP1 and 11.7 mg *Pleos*-DyP4 per *E. coli* culture (9 L), and Reinheitzahl (R_z_) *A*_405_/*A*_280_ ratio of 1.9 and 2.2, respectively.

**Fig. 4:**
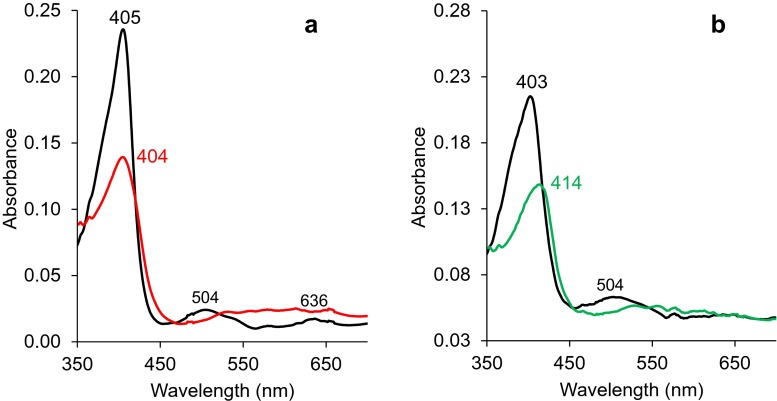
Electronic absorption spectra of *Pleos*-DyP4. **a** Compound I spectrum (*red*) obtained 2 s after addition of 2 eq. of H_2_O_2_ to DyP resting state (*black*) in 10 mM tartrate, pH 3, showing the main Soret band at 404 nm, and disappearance of the maxima at 504 and 636 nm. **b** Compound II-like spectrum (*green*) obtained 2 s after addition of 2 eq. of H_2_O_2_ to DyP resting state (*black*) in 10 mM Tris-HCl, pH 7, showing the main Soret band displacement from 403 to 414 nm, and disappearance of the maximum at 504 nm. Spectra were obtained with an Agilent 8453 diode array UV-visible spectrophotometer

The electronic absorption spectra of the two enzymes were nearly identical, and those of *Pleos*-DyP4 are shown in Fig. 4. The resting state spectrum at physiological pH 3 shows the main Soret band at 405 nm, and two small charge-transfer maxima at 504 and 636 nm, confirming proper incorporation of the cofactor by *E. coli* (Fig. 4a, black line). The two enzymes were able to form reactive compound I, as revealed by the spectral changes after H_2_O_2_ addition, including reduced intensity of the Soret band and disappearance of the above charge-transfer maxima being substituted by several small peaks (Fig. 4a, red line). Compound II seemed to be quickly self-reduced since the corresponding spectrum could not be subsequently observed, and compound I directly returned to the initial spectrum (resting state). However, as reported for other fungal DyPs (Linde et al. 2015a), and illustrated in Fig. 4b for *Pleos*-DyP4, a compound II-like spectrum was observed when the resting enzyme at pH 7 (black line) reacted with H_2_O_2_ resulting in Soret band displacement from 403 nm (as found for resting DyP at pH 7) to 414 nm and disappearance of the 504 nm maximum, among other changes (green line).

For comparison of catalytic properties with DyPs, two MnPs and two VPs from the *P. ostreatus* genome were produced as inclusion bodies, in vitro activated, purified to electrophoretic homogeneity, and characterized, as described elsewhere (Fernández-Fueyo et al. 2014d) (Fig. S1 in the Supplementary Material).

### DyP catalytic properties compared with other *P. ostreatus* peroxidases

**Fig. 5:**
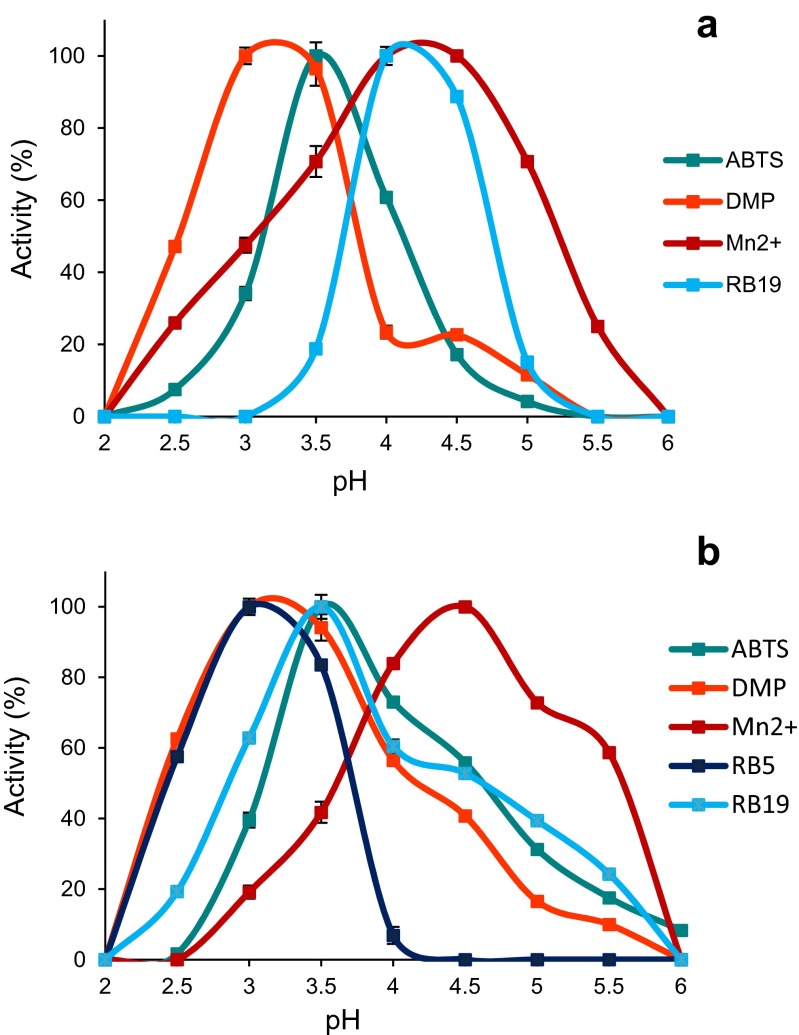
Optimal pH values for the oxidation of ABTS (5 mM), DMP (60 mM), RB5 (15 μM), and RB19 (200 μM) in 0.1 M B&R buffer, and Mn^2+^ (6 mM) 0.1 M tartrate buffer by *Pleos*-DyP1 (**a**) and *Pleos*-DyP4 (**b**) from the *P. ostreatus* genome

Before analyzing their kinetic constants, the optimal pH for oxidation of the different substrates (at saturating concentrations) was determined for *Pleos*-DyP1 and *Pleos*-DyP4 (Fig. 5a, b, respectively). Although *Pleos*-DyP1 showed higher pH optimum for RB19 oxidation than *Pleos*-DyP4, the general pH profiles were similar for both enzymes, with an optimum at pH 3–4 for the phenolic and dye substrates, and a higher optimal pH (4.5) for Mn^2+^, suggesting the involvement of deprotonated acidic residues in the latter activity. In general, no activity was detected over pH 4 in the case of RB5, and over pH 6 for the rest of the DyP substrates. The optimal pH for *P. ostreatus* MnP and VP reactions has been reported elsewhere (Fernández-Fueyo et al. 2014d) being also characterized by their acidic optima.

Table 1 shows the kinetic constants of *Pleos*-DyP1 and *Pleos*-DyP4 (expressed in *E. coli* as active proteins) on five representative substrates, compared with *Pleos*-MnP3, *Pleos*-MnP6, *Pleos*-VP1, and *Pleos*-VP2 (also expressed in *E. coli* but in vitro activated). VA oxidation was also assayed, but, although this nonphenolic substrate was oxidized by VPs, no activity was detected for any of the DyPs and MnPs. The three peroxidase families (DyPs, MnPs, and VPs) differed in their catalytic properties, although the low redox-potential dye ABTS could be efficiently oxidized by all of them. Significant differences were also observed between the two *P. ostreatus* DyPs, as described below.

**Table. 1:** Kinetic constants—*K*_*m*_ (μM), *k*_cat_ (s^−1^), and *k*_cat_/*K*_*m*_ (s^−1^.mM^−1^)—for oxidation of RB19, ABTS, RB5, DMP, and Mn^2+^ by *Pleos*-DyP1 and *Pleos*-DyP4 from the *P. ostreatus* genome, compared with two MnPs and two VPs from the same fungus

		*Pleos*-DyP1	*Pleos*-DyP4	*Pleos*-MnP3	*Pleos*-MnP6	*Pleos*-VP1	*Pleos*-VP2
RB19	*K*_*m*_	45 ± 7	82 ± 13	–^a^	–	3 ± 0.4	16 ± 1
	*k*_cat_	5 ± 0.4	152 ± 13	0	0	11 ± 0.4	8 ± 0.2
	*k*_cat_/*K*_*m*_	113 ± 11	1860 ± 150	0	0	3220 ± 320	495 ± 29
ABTS^b^	*K*_*m*_	779 ± 69	787 ± 160	778 ± 103	1020 ± 80	4 ± 0	12 ± 2
	*k*_cat_	208 ± 8	277 ± 24	222 ± 15	115 ± 5	14 ± 0	9 ± 0
	*k*_cat_/*K*_*m*_	267 ± 15	352 ± 46	285 ± 19	112 ± 4	3600 ± 20	725 ± 36
RB5	*K*_*m*_	–	5.7 ± 0.4	–	–	5.4 ± 0.2	9.6 ± 1.8
	*k*_cat_	0	5.3 ± 0.8	0	0	12.9 ± 0.3	20.3 ± 2.0
	*k*_cat_/*K*_*m*_	0	1080 ± 100	0	0	2380 ± 50	2120 ± 180
DMP^b^	*K*_*m*_	31,100 ± 3800	126 ± 21	59,100 ± 6800	117,000 ± 18,000	54 ± 4	607 ± 57
	*k*_cat_	64 ± 3	268 ± 24	101 ± 7.2	56 ± 6	7 ± 0	17 ± 1
	*k*_cat_/*K*_*m*_	2.1 ± 0	2120 ± 280	1.7 ± 0	0.5 ± 0	122 ± 7	28 ± 1
Mn^2+^	*K*_*m*_	2780 ± 440	286 ± 33	101 ± 12	73 ± 9	98 ± 6	18 ± 2
	*k*_cat_	10 ± 1	56 ± 2	163 ± 5	109 ± 3	185 ± 3	79 ± 2
	*k*_cat_/*K*_*m*_	4 ± 0	196 ± 18	1010 ± 170	1500 ± 100	1900 ± 90	4510 ± 410

Means and 95 % confidence limits (three significant figures) from reactions at 25 °C in 0.1 M tartrate, pH 4.5, and 5 for Mn^2+^ in DyPs and VP and MnP, respectively, and pH 3.5 for RB19, RB5, DMP, and ABTS. Except for RB19 in *Pleos*-DyP1 and *Pleos*-VP1, at pH 4. MnP and VP activities on ABTS, RB5, DMP, and Mn^2+^ are from Fernández-Fueyo et al. (2014d)

^a^dash represents the undetermined *K*_m_ values when no activity was detected

^b^ABTS and DMP oxidation by VPs show a biphasic kinetics enabling calculation of a second set of lower catalytic efficiency constants (not shown)

Only *Pleos*-DyP4 was able to oxidize the high redox-potential RB5, showing a *K*_m_ similar to that of VPs (~5 μM) but with a 2–4-fold lower *k*_cat_ (MnPs show no activity on this compound). In the case of RB19, a typical DyP substrate that we show is also oxidized by VPs; *Pleos*-DyP1 presented *k*_cat_ values similar to *Pleos*-VP1 and *Pleos*-VP2, while the *Pleos*-DyP4 *k*_cat_ is up to 30-folds higher (again MnPs had no activity on this compound). Regarding DMP oxidation, *Pleos*-DyP4 was up to 1000-folds more efficient than *Pleos*-DyP1, due to its lower (250-fold) *K*_m_, and a higher (fourfold) *k*_cat_. *Pleos*-DyP4 was the most efficient enzyme on this phenolic substrate, which is also oxidized not only by VPs but also by the *P. ostreatus* MnPs that belong to the short MnP subfamily (Fernández-Fueyo et al. 2014a). Unexpectedly, the two DyPs from the *P. ostreatus* genome also exhibited Mn^2+^-oxidizing activity, which was especially significant for *Pleos*-DyP4 (near 200 s^−1^.mM^−1^). Both DyPs exhibit lower affinity for Mn^2+^ than the MnPs and VPs, the difference being more dramatic for *Pleos*-DyP1 with a *K*_m_ value 2–3 orders of magnitude higher (mM range). However, the *Pleos*-DyP4 *K*_m_ for Mn^2+^ is only 2–3-folds higher than those of *Pleos*-MnP3, *Pleos*-MnP6, and *Pleos*-VP1.

The above results show that *Pleos*-DyP4 always has higher (1.3–1000-fold) *k*_cat_ values than *Pleos*-DyP1, suggesting that some differences in the heme environment provide this enzyme with a higher redox potential. However, better fitting of the corresponding substrates at the oxidation site can also result in faster electron transfer in *Pleos*-DyP4. For Mn^2+^ and DMP, significantly lower (9–250-fold) *K*_m_ values than those of *Pleos*-DyP1 were also obtained, indicating a better binding of the two substrates by *Pleos*-DyP4.

### pH and thermal stability of DyP isoenzymes

**Fig. 6:**
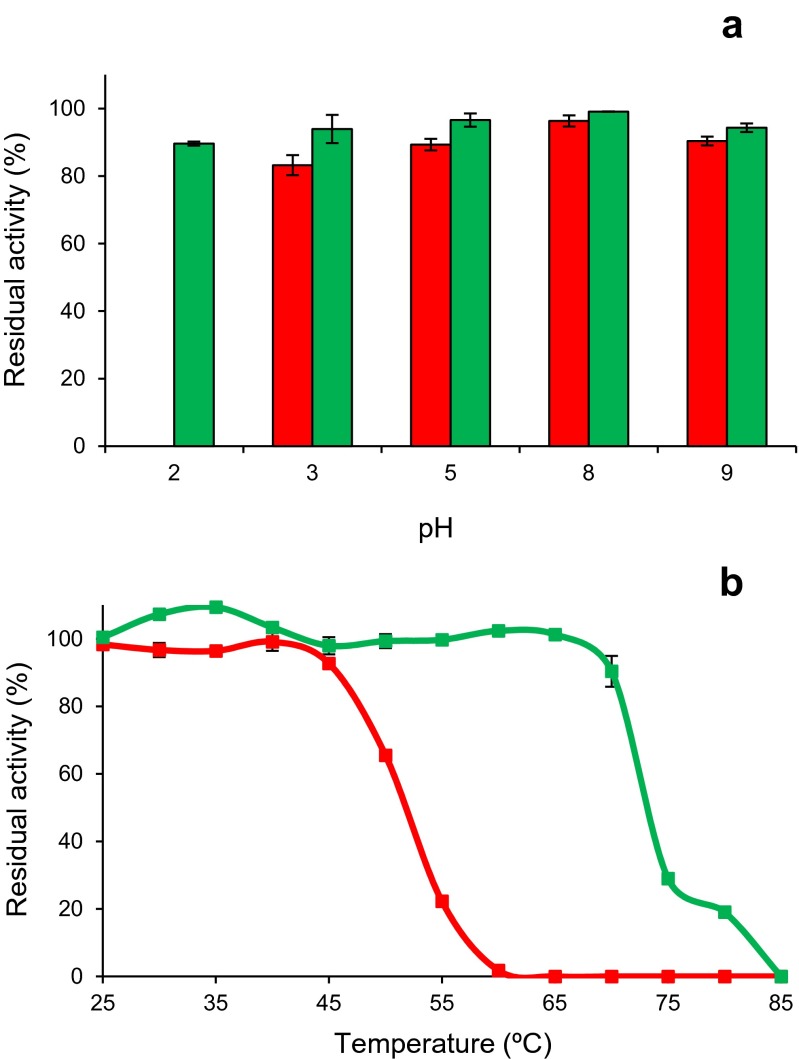
pH and thermal stabilities of *Pleos*-DyP1 (*green*) and *Pleos*-DyP4 (*red*) from the *P. ostreatus* genome. Residual activities were estimated after 24 h incubation in the range of pH 2–9 (in B&R buffer, 4 °C) (**a**) and at different temperatures in the range of 25–70 °C (in 0.01 M tartrate, pH 5) (**b**), and referred to the highest activity measured immediately after adding the enzyme

The pH and thermal stabilities of the two *P. ostreatus* DyPs described above were estimated by incubating the enzymes (0.01 μM concentration) at different pHs (pH 2–9 range) and temperatures (25–85 °C range) and determining the remaining activity. The *P. ostreatus* DyPs are extremely stable at pH 3, maintaining more than 80 % of the initial activity after 24 h incubation (Fig. 6a). However, at pH 2, strong differences appeared between the two enzymes, with *Pleos*-DyP4 still maintaining over 80 % of the initial activity while *Pleos*-DyP1 was very quickly (1 min) fully inactivated. On the other side, both DyPs were also extremely stable at basic pH, maintaining over 80 % of the initial activity after 24 h of incubation at pH 9.

Comparison of the thermal stability of the two DyPs, measured as the residual activity after 10-min incubation (Fig. 6b), showed that *Pleos*-DyP4 was 100 % stable in the 60–70 °C range, while *Pleos*-DyP1 was fully inactivated under the same conditions. From the thermal inactivation profiles, 10-min *T*_50_ values of 52 and 73 °C were estimated for *Pleos*-DyP1 and *Pleos*-DyP4, respectively. The above differences are even higher than those observed for the pH stability.

### Secretomic analysis of peroxidases in glucose, straw, and wood cultures

The secretomes of dikaryotic *P. ostreatus* grown on poplar chips, wheat straw, and glucose (HSA) medium were analyzed by nLC-MS/MS of the total tryptic peptides obtained. Different DyP, MnP, and VP proteins were identified (Table 2) from the presence of unique peptides (at least two per protein and sample) whose sequences were identified by comparison with the available *P. ostreatus* genome.

**Table 2:** Summary of peroxidases in extracellular proteomic analysis of *P. ostreatus* grown in lignocellulose containing media and glucose (HSA) medium (for 14 days at 25 °C) analyzed by nLC-MS/MS

	nLC-MS/MS					Protein properties		
	Score	Coverage (%)^a^	Unique peptides	Total peptides	PSMs	AAs	MW [kDa]	Calculated pI
Poplar chips								
*Pleos*-DyP4	19.15	11.11	5	5	7	504	54.9	6.54
*Pleos*-VP1	158.42	13.61	4	5	47	331	34.6	4.63
*Pleos*-VP2	12.29	5.18	2	2	4	339	35.8	4.56
*Pleos*-VP3	42.15	18.33	2	4	14	360	37.5	4.72
*Pleos*-MnP3	83.59	14.01	5	5	33	335	35.0	4.59
*Pleos*-MnP6	36.68	18.06	6	6	14	360	38.1	5.14
Wheat straw								
*Pleos*-DyP4	67.41	29.96	12	12	21	504	54.9	6.54
*Pleos*-VP1	111.61	20.54	6	6	35	331	34.6	4.63
*Pleos*-VP2	83.11	17.18	5	5	23	339	35.8	4.56
*Pleos*-MnP3	121.04	14.01	5	5	49	335	35.0	4.59
*Pleos*-MnP6	38.23	18.06	6	6	14	360	38.1	5.14
Glucose medium								
*Pleos*-DyP4	98.41	32.74	15	15	33	504	54.9	6.54
*Pleos*-VP1	28.89	15.35	4	4	9	331	34.6	4.63

^a^Coverage, percentage of tryptic peptides identified

*PSM* peptide-spectrum match (indicating the number of scans where a protein is identified), *AAs* amino acid number

*Pleos*-DyP4, *Pleos*-VP1, *Pleos*-VP2, *Pleos*-MnP3, and *Pleos*-MnP6 were identified on both poplar chips and wheat straw, with *Pleos*-VP3 appearing as a sixth peroxidase on poplar chips. In both cases, *Pleos*-VP1 and *Pleos*-MnP3 were the most abundant peroxidases, as revealed by the semiquantitative PSM value, although the abundance order on poplar chips (*Pleos*-VP1 followed by MnP3) was inverted on wheat straw (*Pleos*-MnP3 followed by *Pleos*-VP1). On the other hand, in the HSA medium, only *Pleos*-DyP4 and *Pleos*-VP1 were identified.

*Pleos*-DyP4 was the unique peroxidase of the DyP family identified in the three cultures analyzed, being the 115, 49, and 34 most abundant protein when the whole secretome was analyzed on poplar chips, wheat straw, and HSA cultures, respectively (data not shown). On the other hand, no *Pleos*-DyP1, *Pleos*-DyP2, and *Pleos*-DyP3 peptides were detected in any of the samples.

## Discussion

### Identification of phylogenetically divergent DyPs in the *P. ostreatus* genome

Since their discovery in *B. adusta* (Kim and Shoda 1999), DyPs have been purified and characterized from six additional fungal species, namely *A. auricula*-*judae*, *Exidia glandulosa*, *Irpex lacteus*, *Mycena epipterygia*, *M. scorodonius*, and *Thermomyces albuminosus* (Johjima et al. 2003; Liers et al. 2010, 2013; Salvachúa et al. 2013; Scheibner et al. 2008; Zelena et al. 2009b). Among them, those of *B. adusta* (Sugano et al. 1999), *A. auricula*-*judae* (Liers et al. 2010), *M. scorodonius* (Scheibner et al. 2008), *T. albuminosus* (Johjima et al. 2003), and *I. lacteus* (most probably corresponding to AAB58908 directly submitted to GenBank by Han, 1996, as unidentified *Polyporaceae* peroxidase sequence) (Linde et al. 2015b) had been cloned and sequenced, together with *G. lucidum* (Kung et al. 2014) and *P. ostreatus* (Faraco et al. 2007) DyPs. Among the different basidiomycete DyPs, only those of *B. adusta* and *A. auricula*-*judae* have been characterized from a structural and mechanistic point of view (Linde et al. 2014, 2015a; Strittmatter et al. 2013; Sugano 2009; Yoshida et al. 2011, 2012). However, the availability of sequenced genomes is strongly increasing the number of known DyP sequences in fungi, and up to 37 DyP genes were reported by Floudas et al. (2012) after analyzing 32 fungal genomes of different taxonomic groups. The present study contributes to our knowledge on fungal DyPs by mining the sequenced genome of *P. ostreatus* for enzymes of this new peroxidase family. The molecular models obtained for their structural-functional classification confirm conservation of residues typical of DyPs at both sides of the heme cofactor (Linde et al. 2015b), as well as the surface tryptophan residue recently identified as responsible for high-turnover substrate oxidation by long-range electron transfer (Linde et al. 2015a).

**Fig. 7:**
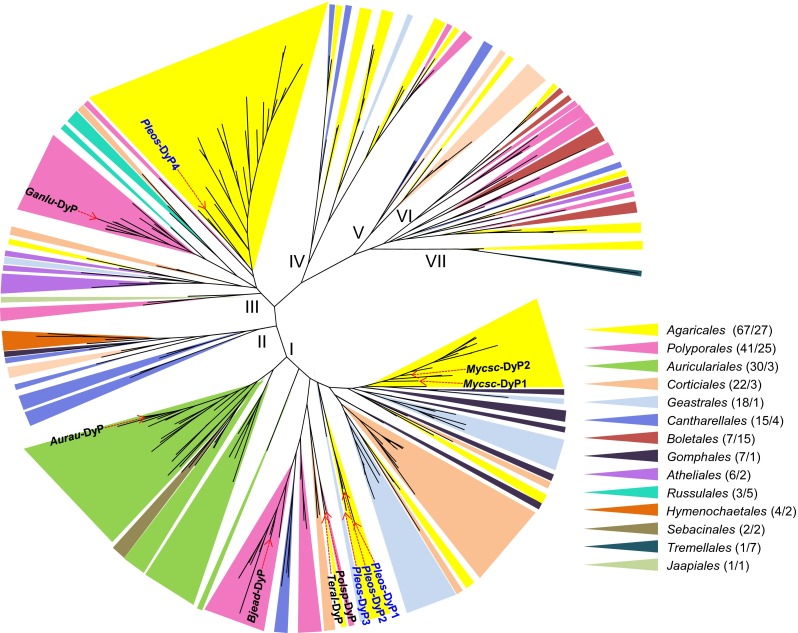
Maximal likelihood phylogram showing the position of the four *P. ostreatus* DyPs with respect to 218 DyP sequences identified in 64 *Agaricomycotina* genomes (among the 114 available at the JGI MycoCosm Portal on 7 December 2014; 45 of them lacking DyP genes and five corresponding to a second sequenced strain). Six GenBank sequences from DyPs of *A. auricula*-*judae* (*Aurau*-DyP, JQ650250), *M. scorodonius* (*Mycsc*-DyP1, CS490662; and *Mycsc*-DyP2, CS490657), *G. lucidum* (*Ganlu*-DyP, ADN05763), unidentified *Polyporaceae* species (*Polsp*-DyP, AAB58908), and *T. albuminosus* (*Teral*-DyP, AAM21606) are also included, as well as *B. adusta* DyP (*Bjead*-DyP, BAA77283; corresponding to JGI *B. adusta* genome protein ID 72253), and their positions indicated on the tree together with those of the *P. ostreatus* DyPs. Subclusters from species of the orders *Agaricales* and *Polyporales* are widespread through the tree, with *P. ostreatus* DyPs in clusters I (*Pleos*-DyP1 to *Pleos*-DyP3) and III (Pleos-DyP4). Although with lower gene numbers, subclusters from *Geastrales*, *Corticiales*, and *Cantharellales* are also widespread. In contrast, DyPs from other orders are restricted to specific clusters: (i) *Auriculariales*, *Gomphales*, and *Sebacinales* to cluster *I*; (ii) *Hymenochaetales* to cluster *II*; (iii) *Atheliales* and *Russulales* to cluster *III*; and (iv) *Boletales* to cluster *VI*. No DyP genes were found in *Dacrymycetales*, *Gloeophylliales,* or *Trechisporales* with 3, 2, and 2 genomes, respectively (order adscription for the different species is from Index Fungorum, http://www.indexfungorum.org). *Colored triangles on the phylogram* show the position of DyPs from the above 14 orders, with the total gene numbers (including six from GenBank) followed by the number of genomes indicated in the legend for each order. *Agaricomycotina* DyP data are from published (Branco et al. 2015; Eastwood et al. 2011; Fernández-Fueyo et al. 2012; Floudas et al. 2012, 2015; Hane et al. 2014; Hori et al. 2014; Janbon et al. 2014; Kohler et al. 2015; Martin et al. 2008; Morin et al. 2012; Ohm et al. 2010; Riley et al. 2014; Rineau et al. 2012; Ruiz-Dueñas et al. 2013) and unpublished genomes available at the JGI MycoCosm Portal (the latter with permission of the project PIs)

To establish the evolutionary histories of the four *P. ostreatus* DyPs, a comparison of all the DyP sequences (a total of 218) from *Agaricomycotina* genomes available to date at the MycoCosm portal (http://genome.jgi-psf.org/programs/fungi) was performed, also including the available DyP sequences (from GenBank) of the above basidiomycete species. The phylogram obtained (Fig. 7) revealed that the *P. ostreatus* DyPs correspond to two largely divergent evolutionary groups. *Pleos*-DyP1, *Pleos*-DyP2, and *Pleos*-DyP3 (together with a few more *Agaricales* DyPs) belong to phylogram cluster I, which includes all the identified DyPs of fungi from the orders *Auriculariales*, *Gomphales*, and *Sebacinales*. Cluster I also includes all the other basidiomycete DyP characterized to date, from *A. auricula*-*judae*, *B. adusta*, *I. lacteus*, *M. scorodonius*, *T. albuminosus*, and *E. glandulosa* (the latter corresponding to one of the sequences of the unpublished genome in the *Auriculariales* subcluster). In contrast, *Pleos*-DyP4 belongs to a large subcluster of *Agaricales* DyPs in the more basal cluster III, predominantly constituted by sequences from *Agaricales* and *Polyporales* (the latter including an hypothetical DyP cloned from *G. lucidum*). Therefore, *Pleos*-DyP4 is the first basidiomycete DyP from a cluster different from cluster I to be characterized. Interestingly, while *Agaricales* present an average of 1.6 DyP genes per genome, which are often located in separate evolutionary clusters (as found in *P. ostreatus*), the opposite tendency was observed in other basidiomycete orders, e.g., in *Auriculariales* with an average of 10.0 DyP genes per genome, all of them belonging to the same evolutionary cluster.

### Heterologous expression of active DyP

An evaluation of the biochemical diversity of the large number of DyP sequences available in basidiomycete genomes is hampered by the difficulties for their heterologous expression, compared with bacterial DyPs that are easily produced in prokaryotic hosts. Similar studies on class II peroxidases have been largely based on *E. coli* expression followed by in vitro activation of the peroxidase protein recovered from inclusion bodies using optimized protocols (Doyle and Smith 1996; Miki et al. 2009; Pérez-Boada et al. 2002).

A similar protocol has been recently developed by Linde et al. (2014) for the in vitro activation of an *E. coli*-expressed *A. auricula*-*judae* DyP, and different variants from site-directed mutagenesis (Linde et al. 2015a). Unfortunately, this protocol is not of general application to other basidiomycete DyPs, most probably because of their structural heterogeneity (e.g., *Pleos*-DyP4 shows less than 40 % identity with the other *P. ostreatus* DyPs).

In the present study, we followed a different approach consisting in optimization of a method for DyP expression as active soluble forms by adding hemin to the *E. coli* culture medium and decreasing the growth temperature to 16 °C after IPTG induction. Similar procedures have been used in the heterologous expression of some bacterial DyPs (Ogola et al. 2009; Santos et al. 2014). Following this strategy, we avoided a costly and time-consuming refolding protocol, although the final purification was more complicated and required three chromatographic steps.

### Remarkable biochemical properties of *P. ostreatus* DyPs

In agreement with their divergent evolutionary origin, *Pleos*-DyP1 and *Pleos*-DyP4 significantly differed in their kinetic and stability properties. Differences in isoenzyme stabilities have been recently reported for *P. ostreatus* MnPs and VPs (Fernández-Fueyo et al. 2014d) and related to differential gene expression under environmental conditions (Fernández-Fueyo et al. 2014b). Similar or even larger differences were observed here, with *Pleos*-DyP4 (i) being over 90 % stable after 24 h at pH 2 (while *Pleos*-DyP1 was fully inactivated in 1 min) and (ii) showing over 20 °C higher 10-min *T*_50_ values (73 °C for *Pleos*-DyP4 compared with only 52 °C for *Pleos*-DyP1).

Compared with other peroxidases, *Pleos*-DyP4 is more thermostable than previously described fungal DyPs (Linde et al. 2014; Salvachúa et al. 2013). However, a recently described bacterial DyP retains 90 % activity after 30 min at 80 °C (Yu et al. 2014). Compared with other enzymes, *Pleos*-DyP4 has higher thermal stability than the peroxidase from the African oil palm tree (*Elaeis guineensis*) that, with a 10-min *T*_50_ of 69 °C, is one of the most thermostable peroxidases described to date (Rodriguez et al. 2002). Both *P. ostreatus* DyPs are stable at pH 3, in agreement with their maximal activity at acidic pH, and the high stability of *Pleos*-DyP4 at pH 2 is similar to that recently reported for a remarkably stable extralong MnP from *Ceriporiopsis subvermispora* (Fernández-Fueyo et al. 2014c).

Concerning catalytic properties, *Pleos*-DyP4 showed high turnover numbers for all the substrates assayed, except for ABTS that was similarly oxidized by the two DyPs, suggesting higher redox-potential than *Pleos*-DyP1, or faster electron transfer due to better substrate fitting at the oxidation site (in agreement with the lack of activity of the latter enzyme on the recalcitrant dye RB5).

However, the most surprisingly catalytic property of *Pleos*-DyP4 (and of *Pleos*-DyP1 in lower degree) is its ability to oxidize Mn^2+^ to Mn^3+^, a reaction that is seen as characteristic of MnP and VP among fungal peroxidases (Ruiz-Dueñas et al. 2009). The *Pleos*-DyP4 turnover for Mn^2+^ (56 s^−1^) was in the same order of those found for the two MnPs (63 and 125 s^−1^) and the two VPs (79 and 185 s^−1^) from the *P. ostreatus* genome, analyzed for comparison. The *Pleos*-DyP4 affinity for Mn^2+^ was also similar (only 2–3-fold higher *K*_m_) to those found for three of the four MnPs/VPs analyzed (the exception being *Pleos*-VP2 that has a fivefold lower *K*_m_). In contrast, *Pleos*-DyP1 has a very low affinity for Mn^2+^, which contributed to its very low catalytic efficiency (50-fold lower than *Pleos*-DyP4). Interestingly, oxidation of Mn^2+^ has been reported for four bacterial DyPs (Brown et al. 2012; Rahmanpour and Bugg 2015; Santos et al. 2014; Singh et al. 2013), albeit with lower turnover (up to 21 s^−1^ for *Amycolatopsis* DyP) than MnPs and VPs. However, this is the first time that Mn^2+^ oxidation is reported for a fungal DyP.

The present study also showed that VPs are able to oxidize RB19, an anthraquinoid dye whose oxidation is often presented as a unique characteristic of DyPs (Sugano et al. 2006). Although they belong to different superfamilies, a significant convergence in catalytic properties has been produced between some DyPs (such as *Pleos*-DyP4) in the CDE superfamily (Goblirsch et al. 2011) and VPs in the catalase-peroxidase superfamily (Zámocký et al. 2015). These enzymes not only adapted very different folds to provide the heme cofactor with an adequate environment, as discussed by Linde et al. (2015b), but they also converged in oxidation of a common range of substrates (from low redox-potential compounds to recalcitrant dyes and Mn^2+^). The specific site for Mn^2+^ oxidation is well characterized in MnPs and VPs (Gold et al. 2000; Ruiz-Dueñas et al. 2009), but, although some crystal structures have been reported for bacterial DyPs containing Mn^2+^ (Singh et al. 2013), more studies are required for a better characterization.

### DyP role in nature: secretomic and other studies

The secretomic studies performed show that *Pleos*-DyP4, and several VPs and MnPs are part of the *P. ostreatus* extracellular proteome during growth on lignocellulosic materials (poplar chips or wheat straw) as the sole C (and N) source, as well as in submerged glucose cultures. This agrees with transcriptomic studies showing widespread expression of DyP genes in forest soils (Kellner et al. 2014). Paradoxically, *Pleos*-DyP4, identified in the different secretomes, is the only *P. ostreatus* DyP that lacks a typical signal peptide. A similar situation has been reported for other basidiomycete oxidoreductases involved in lignocellulose decay, such as *Gloeophyllum trabeum* methanol oxidase (Daniel et al. 2007), suggesting an alternative secretion mechanism (or enzyme release by hyphal autolysis).

Some controversy exists on the natural role of DyPs, which was not reported when the first (fungal) DyP was described as representing a new peroxidase family being able to oxidize substituted anthraquinoid dyes (Kim and Shoda 1999; Sugano et al. 2007). Subsequent studies on some bacterial DyPs described them as lignin-degrading enzymes (Ahmad et al. 2011; Brown and Chang 2014; Rahmanpour and Bugg 2015). However, the evidence is scarce and largely based on modification of uncharacterized lignocellulosic materials and oxidation of phenolic dimers (Ahmad et al. 2010, 2011; Brown et al. 2012; Rahmanpour and Bugg 2015). Even if some fungal, DyPs are also able to oxidize veratryl alcohol and nonphenolic lignin model dimers (representing the main substructures in the polymer) (Liers et al. 2010, 2013; Linde et al. 2014); their activity is too low to play a relevant role in the initial attack to the lignin polymer in nature (Linde et al. 2015b).

The present study reveals the evolutionary and biochemical diversity of DyPs in the *P. ostreatus* genome. Moreover, it shows that one of them (*Pleos*-DyP4) shares catalytic properties with VP, whose ligninolytic ability has been recently demonstrated (Fernández-Fueyo et al. 2014d), including oxidation of recalcitrant RB5 and Mn^2+^. A rather unique property of *P. ostreatus*, evidenced in the present study, is the secretion of three different peroxidase types being able to oxidize Mn^2+^. Their similarities with ligninolytic peroxidases, together with their simultaneous secretion in lignocellulose cultures and presence in natural habitats, suggest that DyPs could participate in lignocellulose degradation by oxidizing lignin-derived compounds or modified lignin, contributing to the genesis of lignin-derived organic matter in soils.

## Electronic supplementary material

ESM 1 (PDF 208 kb)
