# Supplementary material for: Description of the first fungal dye-decolorizing peroxidase oxidizing manganese(II)
Source: Appl Microbiol Biotechnol. 2015 May 13;99(21):8927–42. doi: 10.1007/s00253-015-6665-3 (PMC4619462; doi:10.1007/s00253-015-6665-3)
Supplement: Supplementary file 1 — (PDF 208 kb) [file 253_2015_6665_MOESM1_ESM.pdf]

Applied Microbiology and Biotechnology

SUPPLEMENTAL TABLE AND FIGURE

Description of the first fungal dye-decolorizing peroxidase oxidizing manganese(II)

Elena Fernández-Fueyo, Dolores Linde, David Almendral, María F. López-Lucendo, Francisco J. Ruiz-Dueñas, Angel T. Martínez

Centro de Investigaciones Biológicas, CSIC, Ramiro de Maeztu 9, E-28040 Madrid, Spain

Corresponding author:

A.T. Martínez

E-mail: ATMartinez@cib.csic.es

Phone: +34 918373112 (ext 4407)

Fax: +34 915360432

http://www.cib.csic.es/lignina/lignina_en.html

Table S1. Amino-acid sequence identities between the four DyPs from the *P. ostreatus* genome (JGI protein references included). The number of residue pairs considered for each comparison is shown after the identity percentage.

	Pleos-DyP4	*Pleos*-DyP3	*Pleos*-DyP2	*Pleos*-DyP1
Pleos-DyP1 (62271)	39% (190)	63% (298)	67% (323)	100% (497)
Pleos-DyP2 (1092668)	39% (191)	74% (350)	100% (488)	-
Pleos-DyP3 (52170)	39% (188)	100% (474)	-	-
Pleos-DyP4 (1069077)	100% (504)	-	-	-

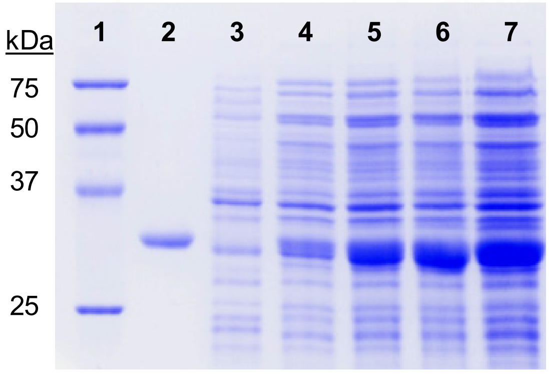

Fig S1 SDS-PAGE during *Pleos*-MnP3 expression in *E. coli*, and after *in vitro* activation and purification. *Lanes 3-7*, time-course of *Pleos*-MnP3 overexpression (as inclusion bodies) in IPTG-induced *E. coli* cultures (total proteins 0, 1, 2, 3 and 4 h after induction, respectively). *Lane 1*, purified *Pleos*-MnP3 in a single chromatographic step, after the *in vitro* activation of protein from *E. coli* inclusion bodies. *Lane 1*, standards (Fernández-Fueyo et al. 2014d).
